# Hydrogen-driven, ATP-dependent biocatalytic reduction of carboxylic acids under non-explosive conditions

**DOI:** 10.1039/d5gc03751d

**Published:** 2025-11-05

**Authors:** Marianna Karava, Qian Liang, Elske van der Pol, Margit Winkler, Robert Kourist

**Affiliations:** a acib GmbH Krenngasse 37 8010 Graz Austria; b Institute for Molecular Biotechnology, TU Graz Petersgasse 14 8010 Graz Austria kourist@tugraz.at; c Institute of Organic Chemistry, TU Graz Stremayrgasse 9 8010 Graz Austria

## Abstract

The reduction of carboxylic acids to alcohols is a key chemical transformation. We present a whole-cell biocatalyst employing carboxylic acid reductases and H_2_ for cofactor regeneration. H_2_ as a reductant ensures high atom and mass efficiency, enabling a more sustainable route to alcohols. Notably, we showcase that the reaction proceeds under O_2_-limited conditions, demonstrating potential for safer bioprocesses.

## Abstract 2

The reduction of carboxylic acids to alcohols in a hydrogen-driven whole-cell biocatalyst employing carboxylic acid reductases ensures high atom and mass efficiency, enabling a potentially more sustainable route for the synthesis of alcohols.

## Graphical abstract

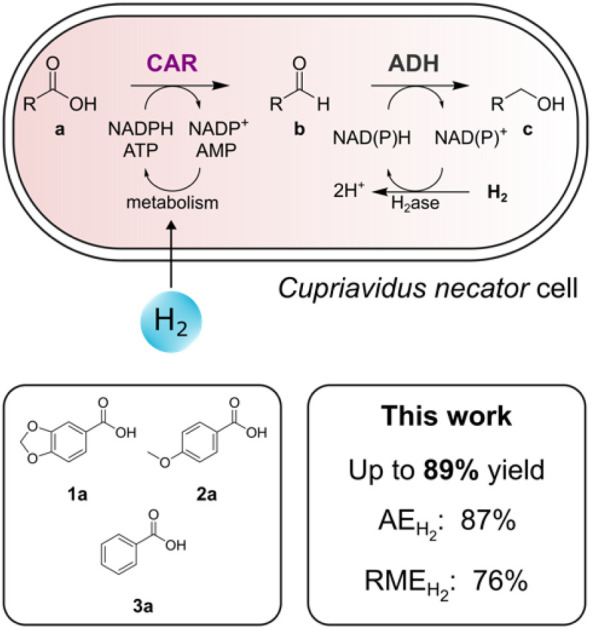

**Green foundation**

1. This work introduces a novel biocatalytic process for the reduction of carboxylic acids to alcohols using molecular hydrogen as a clean and efficient reductant. The study makes two significant contributions: (i) it demonstrates for the first time the use of molecular hydrogen in ATP-dependent biotransformations, and (ii) the reaction operates under oxygen-limited conditions, enhancing process safety and aligning with key principles of green chemistry.

2. The approach achieved product yields of up to 89%. By employing molecular hydrogen as the reductant, the process exhibits substantially improved atom and mass efficiency compared to conventional glucose-driven systems, offering a more sustainable alternative.

3. Future work will focus on scaling up the reaction and optimizing process conditions. A key direction will be integrating biomass generated from gaseous feedstocks as a biocatalyst source, enabling a fully hydrogen-driven reduction system and further advancing the overall sustainability of the approach.

## Introduction

**Fig. 1:**
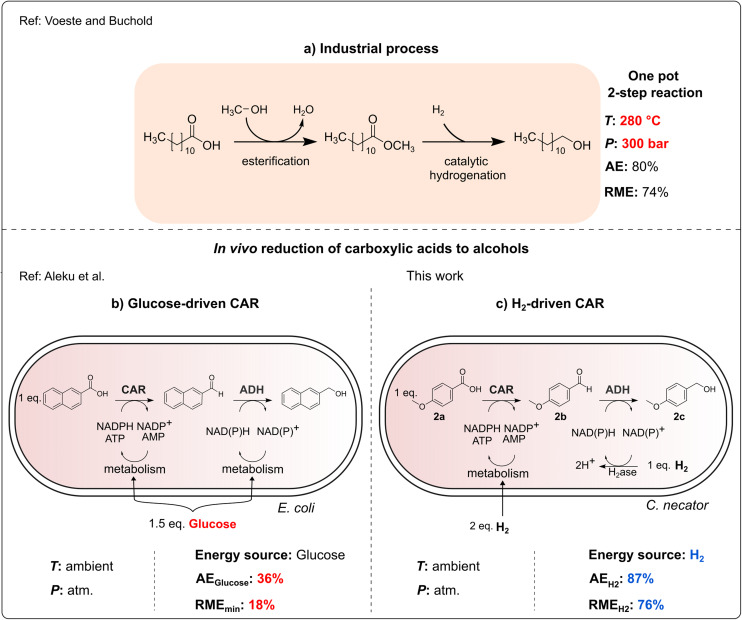
Comparison of different processes for the reduction of carboxylic acids to alcohols. (a) Industrial process for production of fatty alcohols.^12^ Atom economy (AE) is calculated for the case of C_12_H_26_O. RME is reported by Constable *et al.* for the hydrogenation reaction.^10^ Whole-cell biotransformation using (b) *E. coli* recombinant cells and glucose for cofactor regeneration.^7^ RME is calculated from literature data assuming that d-glucose is fully consumed in the reaction.^7^ (c) *C. necator* and H_2_ for cofactor regeneration. RME is calculated from HPLC-yields obtained in this study assuming that H_2_ is consumed in stoichiometric amounts. Calculations for the AE and RME are available in the SI (Table S3).

The reduction of carboxylic acids to alcohols is a highly important transformation in industrial chemistry. For instance, fatty alcohols are widely used in detergents, cleaning agents and cosmetics, with a yearly production of 3 million tonnes.^1^ Production of alcohols from carboxylic acids usually requires esterification or transesterification, respectively, followed by metal-catalyzed hydrogenation at elevated temperatures (*e.g.* 280 °C) and pressures (*e.g.* 300 bar).^1^ Alternative chemical methods for carboxylic acid reduction with metal hydrides as reductants require very low temperatures (−78 °C), which is very energy-consuming.^2^ Therefore, direct enzymatic reduction of carboxylic acids has gained considerable attention.^1,3–6^ The reaction can be easily accomplished by carboxylic acid reductases (CARs) either cell-free or in whole-cell biocatalysis mode.^7^ Interestingly, nature uses a route similar to the metal-catalyzed hydrogenation. CARs employ ATP and NADPH as stoichiometric cosubstrates. The activation of the carboxylic acid with ATP results in an acyl adenylate intermediate, which is then reduced by NAD(P)H to form the corresponding aldehyde.^8^ In addition to the cofactor dependence, CAR activity necessitates post-translational modification *via* phosphopantetheinyl transferase.^8^ Therefore, the functional production of this important biocatalyst in bacteria is a significant accomplishment. While *in vitro* CAR mediated biotransformations lead to the formation of aldehydes, in whole-cell biocatalysts, alcohol dehydrogenases (ADH) of the host cell tend to reduce the aldehyde further to the respective alcohol (Fig. 1). This enzymatic cascade may lead to the formation of a mixture of the aldehyde and alcohol, depending on the availability of reducing equivalents.^7^

In the application of CAR in whole-cell systems, addition of glucose or other electron-rich donor molecules for the recycling of the stoichiometric cofactors NAD(P)H and ATP leads to a rather low atom economy (AE) of the process. AE is a measure of the efficiency of a chemical reaction, with regard to the ratio of the starting materials that are utilised in the formation of the desired product, as opposed to those that are discarded as waste.^9^

AE alone does not capture the actual efficiency of a process. Reaction Mass Efficiency (RME) offers a complementary metric, taking into account not only how much of the reactants are incorporated into the product but also the actual mass of reagents used. A low RME indicates that a large portion of the input materials contribute to waste.^10^

Glucose-driven whole-cell reduction has a significantly low AE and RME. Assuming that two molecules of NAD(P)H can be regenerated per molecule of glucose, the theoretical AE for the whole-cell reduction of a similar compound is only 37%.^7,11^ Additionally, the minimum RME is further diminished to 18% due to the relatively high molecular weight of glucose, making the overall process less efficient and more waste-intensive.

Hydrogen, on the other hand is a sustainable electron donor that enables efficient recycling of cofactors without by-product formation. Assuming that one hydrogen molecule is required for the formation of one cofactor molecule, we calculated for the substrate *p*-anisic acid (2a) an AE of 87% (Table S3). Since hydrogen is used in small molar quantities and generates no waste mass, the RME of the hydrogen-driven process is also substantially improved (RME 76%), making it both atom- and mass-efficient. Notably, this compares favourably to the industrial process for fatty alcohol production, which proceeds *via* chemical esterification and hydrogenation steps over a time span exceeding 12 hours and has a theoretical AE in the scale of 80%. The reported RME values for esterification and hydrogenation are 68% and 74%, respectively, indicating that the hydrogen-driven biotransformation is on par with established industrial methods in terms of material efficiency.^10^

Coupling of hydrogenases to oxidoreductases that require NAD(P)H has been successfully demonstrated with free as well as with immobilized enzymes.^13–17^ Interestingly, in some cases the proton transfer can be accomplished without nicotinamide cofactor.^15^ However, these *in vitro* systems are limited to electron-transfer processes and cannot be applied to ATP-dependent biotransformations.^15,18^ This issue could be circumvented by employing chemolithoautotrophic organisms as whole-cell biocatalysts. Ni *et al.* demonstrated the feasibility of biocatalytic hydrogenation of carboxylic acids *in vivo* by the native aldehyde oxidoreductase and hydrogenase of the thermophile *Pyrococcus furiosus*.^3^ The authors achieved complete reduction of 10 mM carboxylic acid to alcohol within 24 hours using 150 mg mL^−1^ cell wet weight (cww).^3^ Despite the considerable synthetic interest this approach offers, it is constrained by a number of factors: firstly, the thermophile bacterium *P. furiosus* is an obligate anaerobe, which presents a challenge in the laboratory.^19^ Secondly, the organism lacks genetic amenability compared to model organisms and grows optimally at high temperatures which can be inhibitory for many enzymes.^20^ In contrast, the aerobic hydrogen-oxidizing bacterium *Cupriavidus necator* offers its capacity to reach high cell densities, and the availability of genetic tools that allow efficient overexpression of heterologous genes.^21–23^ The application of *C. necator* in whole-cell H_2_-fueled biotransformations has already been demonstrated for oxidoreductases. We have previously shown that *C. necator* recombinant cells can be applied for the activation of double bonds using an NADPH-dependent ene-reductase.^24^ Oda *et al.* demonstrated the reduction of ketones by *C. necator* whole cells producing NADH-dependent alcohol dehydrogenases.^25^ More recently, Jämsä *et al.* developed a whole-cell system for the reduction of d-xylose to xylitol by producing the NADH-dependent d-xylose reductase.^26^ The highest xylitol productivity reached was as high as 0.7 g L^−1^ h^−1^, underlining the potential of *C. necator* for biotechnological production.^26^

The chemolithoautotrophic bacterium offers the metabolic machinery for the provision of both cofactors for the CAR reaction. *C. necator* assimilates CO_2_*via* the Calvin–Benson–Bassham (CBB) cycle. Interestingly, this process requires both NADPH and ATP that are generated chemolithoautotrophically using H_2_ as electron donor and O_2_ as electron acceptor. NADH and NADPH are provided directly by the soluble hydrogenase and the transhydrogenase located in the cytoplasm, respectively. The membrane-bound hydrogenase (MBH) contributes to the proton gradient across the inner membrane directly by releasing two protons on the periplasmic side of the inner membrane, and indirectly by reducing ubiquinone to ubiquinol, whose oxidation in the respiration chain adds to the membrane gradient. The membrane gradient created by MBH activity adds to respiratory oxidation of NADH and fuels ATP synthesis by the ATPase. MBH thus contributes to the utilization of reduction equivalents from hydrogen for ATP synthesis (Fig. S1).^21,27^ Chemolithoautotrophic growth of *C. necator* depends on the availability of H_2_, CO_2_ and O_2_.^28^ Usually, hydrogen as the least soluble gas is provided in excess, with a typical ratio of H_2_ : CO_2_ : O_2_ of (7 : 1 : 1). Unfortunately, gas mixtures containing H_2_ and O_2_ with more than 4% each are explosive and highly problematic for technical application.^29^ Thus, we were interested to explore whether cultivation of *C. necator* cells expressing the genes of CAR, and the hydrogen-driven whole-cell biotransformations would be possible under a gas atmosphere containing less than 4% O_2_. Combustion and flame propagation of hydrogen require a limiting (minimum) oxygen concentration (LOC) in the range of 4–6%.^30^ Therefore, hydrogen-driven processes operated below this threshold can be handled at the technical scale with moderate risk, provided that standard process-safety measures are implemented (*e.g.*, ATEX-compliant systems as defined in European Union guidelines). Tang *et al.* successfully cultivated *C. necator* under O_2_-limited conditions (3% O_2_) for the production of polyhydroxybutyrate (PHB) at laboratory scale reaching 0.47 g L^−1^ dry cell weight and 41.5% PHB content.^31^ These results demonstrate both performance and safe process operation under non-explosive conditions. The contribution of MBH to the membrane gradient and thus to ATP synthesis raised the expectation that also an ATP-dependent enzyme reaction should be possible under non-explosive conditions. The direct reduction in *C. necator* offers very mild reaction conditions and saves reaction steps. The objective of our study was to address two significant research questions. First, the feasibility of the functional production of CARs in *C. necator* and second, the efficiency of the reduction of carboxylic acids by the whole-cell biocatalyst under O_2_ limited conditions.

## Results and discussion

To investigate the feasibility of producing carboxylic acid reductases from fungi and plants in *C. necator*, we expressed three car genes from *Mycobacterium marinum* (*Mm*CAR; WP012393886.1), *Dichomitus squalens* (*Ds2*CAR; XP007365347.1) and *Neurospora crassa* (*Nc*CAR; XP955820.1).^6,32,33^ For activation of the CAR, we coexpressed the gene of the *Escherichia coli* phosphopantetheinyl transferase (*Ec*PPTase; ACA78686.1) in an artificial operon under the control of the constitutive promoter P_j5_.^34^ The strains were cultivated in complex media supplemented with fructose as carbon source while the whole-cell biotransformation was performed in buffer supplemented with fructose for cofactor regeneration. As case study substrates, we selected phenolic carboxylic acids whose corresponding aldehydes and alcohols are widely applied in the food and cosmetics industries as aroma and flavour ingredients. For instance, piperonal (heliotropin) is a valuable aromatic aldehyde commonly used in fine fragrances and as a flavouring compound.^35^ We were pleased to find that all three CARs showed activity towards 1a–3a. Among the three different CARs, the *Nc*CAR containing biomass exhibited the highest catalytic activity, with a conversion of 87%. In contrast, the conversions obtained for the two other CARs remained relatively low (Table 1). Notably, the cells had formed the alcohols 1c–3c after 16 h. The corresponding primary products 1b–3b were not detected, indicating that the aldehydes were further reduced by the endogenous ADH from *C. necator*.^36^ The overreduction of the formed aldehyde to alcohol is a comparable result to the direct hydrogen-driven reduction reported by Hollmann *et al.*^3^ It should be noted that in some cases mass imbalances were observed, attributed to the volatility of the obtained products.

**Table 1:** Direct fructose-driven reduction of carboxylic acids in whole-cells of *C. necator* produced under the control of the constitutive promoter P_j5_ in a fructose-rich medium^a^

Entry	Enzyme	Substrate (a)	Conversion^b^ [%]	Yield^b^ [%]	
				Aldehyde (b)	Alcohol (c)
1	*Nc*CAR	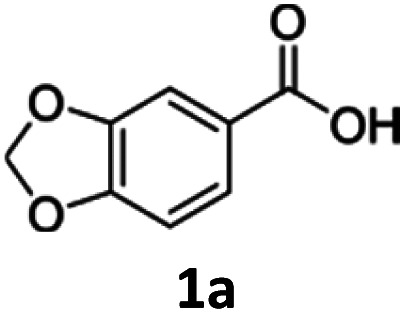	87 ± 3	n.d.	81 ± 1
2	*Mm*CAR		<10	n.d.	<10
3	*Ds2*CAR		12 ± 4	n.d.	12 ± 0
4	*Nc*CAR	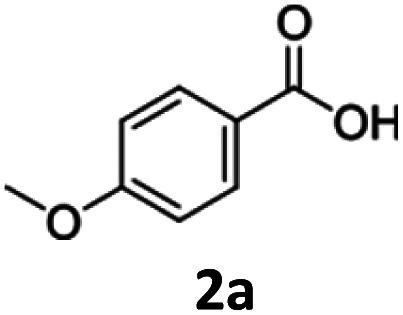	79 ± 8	n.d.	79 ± 6
5	*Mm*CAR		<10	n.d.	<10
6	*Ds*2CAR		<10	n.d.	<10
7	*Nc*CAR	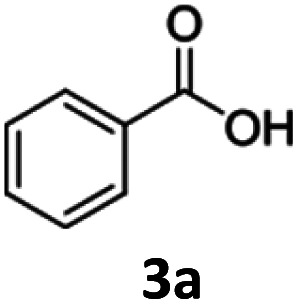	72 ± 3	n.d.	61 ± 2
8	*Mm*CAR		<10	n.d.	<10
9	*Ds*2CAR		<10	n.d.	<10

a Conditions: 2.5 mM **a**, 0.2% d-fructose, 26 mg mL^−1^ CDW, potassium phosphate buffer (0.2 M, pH 7.4), 28 °C, 16 h.

b % conversion and HPLC-yield of aldehyde and alcohol, respectively, was determined by HPLC; n.d. = not detected.

**Fig. 2:**
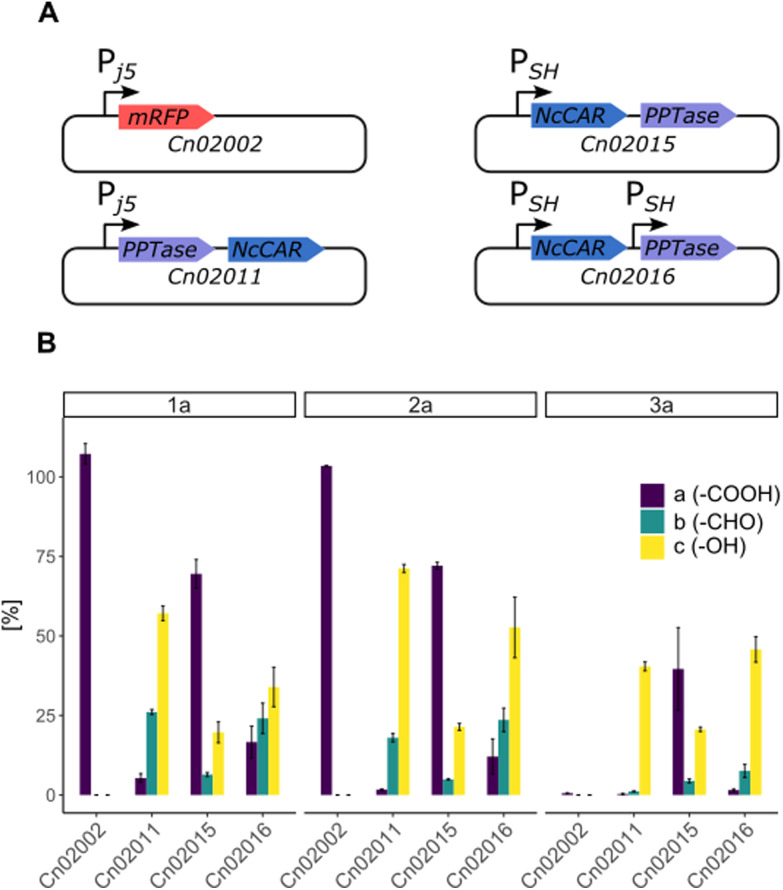
H_2_-driven bioreduction of carboxylic acids catalyzed by engineered *C. necator* strains producing *Nc*CAR. (A) Genetic constructs designed for the production of *Nc*CAR. Cn02002 (P_j5__RFP): control strain, Cn02011: expression of *Nc*CAR under the control of P_j5_ promoter, Cn02015: expression of *Nc*CAR under the control of P_SH_ promoter, Cn02016: expression of *Nc*CAR under the control of P_SH_. (B) Abundance of substrate a (carboxylic acid) and products b (aldehyde) and c (alcohol) after 16 h. Biotransformations were performed under an atmosphere of H_2_ : CO_2_ : O_2_ (7 : 1 : 1). Conditions: 2.5 mM a, 18 mg mL^−1^ CDW, potassium phosphate buffer (0.2 M, pH 7.4), 28 °C. [%] corresponds to remaining substrate and product yields. Error bars represent standard deviation (*n* = 3).

Cultivation under autotrophic conditions (H_2_/CO_2_/O_2_ atmosphere), or, interestingly, in defined medium containing fructose–glycerol–nitrogen (FGN) leads to significantly higher hydrogenase production than cultivation on fructose as sole carbon source.^37,38^ FGN medium enables a substrate shift from the preferentially used carbon source (fructose) to the less-preferred glycerol, resulting in derepression of the *hox* regulon that controls the expression of the hydrogenase genes.^39^ As cultivation on FGN medium poses no explosion risk and favours hydrogenase production, we selected this approach for the production of the recombinant cells under safe conditions. We envisioned that the native soluble hydrogenase promoter (P_SH_) should be active both under heterotrophic conditions with glycerol as carbon source, and under chemolithoautotrophic conditions. Therefore, we compared the production of *Nc*CAR in an artificial operon with *Ec*PPTase, under the control of the P_j5_ and P_SH_ promoters, respectively (strains Cn02011 and Cn02015, Fig. 2A). Additionally, both genes were expressed separately under the control of the P_SH_ promoter (strain Cn02016, Fig. 2A). Small scale (5 mL) biotransformations were performed in gas tight vessels filled with a gas mixture of H_2_ : CO_2_ : O_2_ (7 : 1 : 1). To our pleasure, *Nc*CAR producing strains proved to be highly active in the hydrogen-driven reduction of several carboxylic acids. Strain Cn02011 showed better performance compared to Cn02015 and Cn0216 (Fig. 2B). Notably, in hydrogen-driven biotransformations, a mixture of aldehyde and alcohol products was obtained, whereas in fructose-driven biotransformations, alcohol was the only product. This suggests that the expression of ADHs varies depending on the cultivation conditions, and that the product formation can be directed toward the aldehyde, similar to the successful inactivation of genes of alcohol dehydrogenases in *E. coli*.^40^ The highest yield in analytical scale biotransformations, referred to as HPLC-yield, of ∼89% was obtained for the reaction with substrate *p*-anisic acid (2a), while for piperonylic acid (1a) and benzoic acid (3a) the measured yields were ∼83% and ∼53% respectively. We noted some losses due to the high volatility of the products, particularly the aldehyde. Moreover, *C. necator* can degrade 3a and utilize it as a carbon source.^41^ A control strain (Cn02002) not expressing the *car* genes completely consumed the substrate 3a.

We then determined the specific activities of strains Cn02011 and Cn02016 for each substrate in H_2_ promoted biotransformations (Table 2). Limiting factors can be assumed to be on the one hand the production of the enzyme and its posttranslational modification. It is known that transport of substrates and products across the cell membrane can be a limiting factor of *C. necator* whole-cell biocatalysts.^24^ On the other hand, supply of cofactor is a crucial aspect. Metabolic supply of NADPH and ATP can be either limited by insufficient activity of the enzymatic pathways leading to these cofactors, or by limitations of the hydrogen mass transfer across the gas–liquid interface. Even though strain Cn02011 had higher specific activity than Cn02016, SDS-PAGE analysis showed a stronger band for the CAR in the latter (Fig. S1). This indicates incomplete phosphopantetheinylation of the apo-CAR and points out an avenue for future optimization of the system. Incomplete maturation of CAR has been observed in *E. coli*.^42^ The specific activities range from 0.2 U g^−1^ to 0.35 U g^−1^ CDW corresponding to a space time yield (STY) of 73–146 µmol L^−1^ h^−1^. These values compare favourably to 0.0043 U g^−1^ cells wet weight reported by Hollmann and coworkers, as well as with other *in vivo* systems that employ CARs reported in literature.^3^ For example, Ahsan *et al.* reported STYs in the range of 0.2–1 mmol L^−1^ h^−1^ for the production of α,ω-diols.^43^ However, product titers achieved here remain low compared to glucose driven *E. coli* systems that performed the reduction of piperonylic acid employing *Nc*CAR. Schwendenwein *et al.*, reported STY of 10 mmol L^−1^ h^−1^ for the reduction of 1a to 1b.^6^ These results underscore the importance of further optimization of enzyme production and post-translational modification in *C. necator* to enhance biocatalytic performance, as the present state of technology of *C. necator* is still far from the efficiency of industrial chemical processes.

**Table 2:** Comparison of strains with different promoters in H_2_-driven biotransformations. Specific activity is related to the cell wet weight (CDW)^a^

Entry	Strain	Substrate (a)	Conversion^b^ [%]	Yield aldehyde^b^ (b) [%]	Yield alcohol^b^ (c) [%]	Total product yield [%]	Specific activity^c^ (U g^−1^_CDW_)	STY^d^ (µmol L^−1^ h^−1^)
1	Cn02011	1a	95 ± 1	26 ± 1	57 ± 2	83 ± 1	0.35 ± 0.02	128.9 ± 3.9
2		2a	98 ± 1	18 ± 1	71 ± 1	89 ± 1	0.29 ± 0.03	146.6 ± 3.4
3		3a	>99	<5	40 ± 1	41 ± 1	0.21 ± 0.01	73.2 ± 2.1
4	Cn02016	1a	84 ± 4	24 ± 5	34 ± 6	58 ± 9	0.22 ± 0.01	97.9 ± 15.2
5		2a	88 ± 4	24 ± 4	53 ± 10	77 ± 5	0.18 ± 0.01	134.4 ± 19.0
6		3a	98 ± 1	8 ± 2	46 ± 4	53 ± 5	0.15 ± 0.02	90.0 ± 8.4

a Conditions: 2.5 mM **a**, 18 mg mL^−1^ CDW, potassium phosphate buffer (0.2 M, pH 7.4), 28 °C, H_2_ : CO_2_ : O_2_ (7 : 1 : 1) 16 h.

b % conversion and HPLC yield of aldehyde and alcohol, respectively, was determined by HPLC (*n* = 3).

c Determined after 1 hour of reaction (*n* = 3).

d STY = space time yield; n.d. = not detected.

Following, we sought to investigate the effect of the gas composition on the efficiency of the biotransformations. For this purpose, we carried out reactions with different gas mixtures of H_2_ : CO_2_ : O_2_. We focused on varying the H_2_ and O_2_ concentrations in order to create two different conditions: explosive (O_2_ > 4%) and non-explosive (O_2_ < 4%). For the explosive conditions, a gas atmosphere consisting of H_2_ : CO_2_ : O_2_ in a ratio of 7 : 1 : 1 was used, whereas for the non-explosive conditions, the respective gas composition was 8 : 1 : 0. It should be noted that trace amounts of oxygen were still present in the system. Additionally, we performed reactions in which the headspace was filled with atmospheric air in order to assess the reducing activity of the cells fuelled by stored reducing equivalents.

**Fig. 3:**
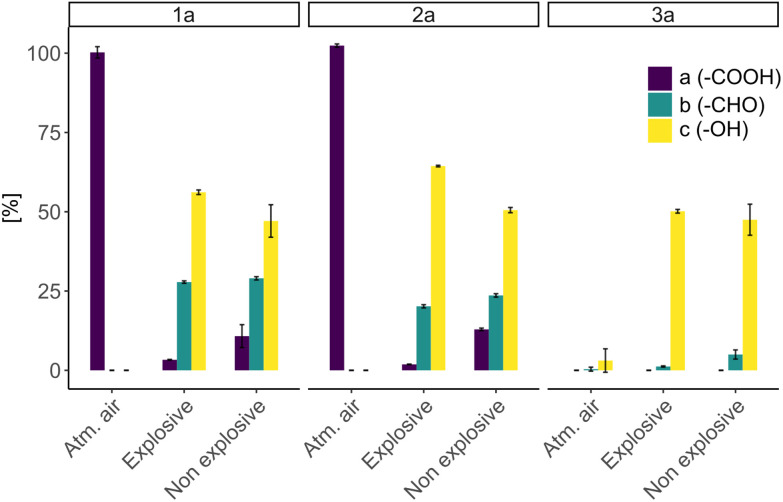
H_2_-driven whole-cell bioreduction of carboxylic acids catalyzed by strain Cn02011 in different gas atmospheres. Explosive conditions H_2_ : CO_2_ : O_2_ (7 : 1 : 1), Non explosive conditions H_2_ : CO_2_ : O_2_ (8 : 1 : 0). Bioreduction of 1a, 2a and 3a respectively. Conditions: 2.5 mM a, 18 mg mL^−1^ CDW, potassium phosphate buffer (0.2 M, pH 7.4), 28 °C. [%] corresponds to remaining substrate (a) and product yields (b, c) respectively. Error bars represent standard deviation (*n* = 3).

In the control reactions under atmospheric air (no H_2_ present), no product formation was observed, which clearly shows that the observed biotransformations are H_2_-driven. Under atmospheric air though, consumption of substate 3a was observed without product formation which can be attributed to the ability of *C. necator* to metabolize benzoic acid.^41^ Oxygen is essential for the generation of ATP *via* the respiratory chain. Therefore, we anticipated that oxygen limitation (well below 4%) would lead to a dramatic decrease in the efficiency of the biotransformation. To our surprise, comparison of whole-cell biotransformations showed that both gas mixtures resulted in similar conversions, with only slightly reduced product yields under oxygen limited conditions (Fig. 3). We attribute the sustained activity to trace amounts of oxygen remaining in the system. Overall, these results indicate that the biotransformation can be performed under O_2_-limited and hence non-explosive conditions as well.

To demonstrate the synthetic application of the whole-cell biotransformation, we scaled up the bioreduction of 1a to 50 mg in 50 mL reaction. The biotransformation was conducted under a gas atmosphere of H_2_ : CO_2_ : O_2_ (7 : 1 : 1) to ensure optimal conditions for the process. As the substrate concentration was increased compared to the analytical scale, to ensure maximal conversion, the biotransformation was performed in two steps using two batches of cells. After 54 hours the reaction reached 55% yield (Fig. S2). The isolated yield was 32% corresponding solely to **1c** as confirmed by NMR (Fig. S3). While this shows the feasibility of conducting the gas-driven reduction in multi-mg scale, the lower volumetric productivity compared to analytical scale is an indication that the mass transfer across the gas–liquid interface is a critical aspect of the up-scale.

## Conclusions

In conclusion, we have demonstrated a novel biocatalytic route for the direct hydrogenation of carboxylic acids to alcohols using *C. necator* cells and hydrogen as reductant. This is the first report of the functional production of CARs in *C. necator*, and it demonstrates the potential of recombinant cells in whole-cell biotransformations, with conversions as high as 89%. Very importantly, our findings illustrate the capacity of this biocatalytic system to support NAD(P)H and ATP dependent enzymes even under oxygen limited conditions, thereby avoiding the safety hazards associated with explosive gas mixtures. By combining high atom and mass efficiency, this work showcases the biocatalytic potential of the hydrogen oxidizing bacterium *C. necator* in chemical synthesis. Yet aspects such as water consumption, the formation of aqueous waste and isolation of alcohol from solvents are challenges that need to be addressed during the further process optimization to develop a truly sustainable process.

## Author contributions

M. K. coordinated, planned and performed part of the experiments. She also did the data analysis and prepared the manuscript. Q. L. measured the initial rates, performed reactions under non explosive conditions and did the preparative scale reaction. E. P. did the workup of the preparative scale reaction and the NMR analysis. M. W. provided the CAR genes and the analytics. She also participated in the interpretation of the data. R. K. provided funding, supervised the work, participated in the data interpretation and prepared the manuscript. All authors reviewed and approved the manuscript.

## Conflicts of interest

There are no conflicts to declare.

## Supplementary Material

GC-027-D5GC03751D-s001

## Data Availability

The data that supports the findings of this study (*e.g.* protein sequences, spectra *etc*) can be found in the supplementary information (SI). Supplementary information is available. See DOI: https://doi.org/10.1039/d5gc03751d. Source data have been deposited in a public GitHub repository (https://github.com/mka-p/H2CAR.git).
