# Supplementary material for: Hydrogen-driven, ATP-dependent biocatalytic reduction of carboxylic acids under non-explosive conditions
Source: Green Chem. 2025 Nov 5;27(47):15049–55. doi: 10.1039/d5gc03751d (PMC12605835; doi:10.1039/d5gc03751d)
Supplement: GC-027-D5GC03751D-s001 [file GC-027-D5GC03751D-s001.pdf]

Supplementary Information (SI) for Green Chemistry.
This journal is © The Royal Society of Chemistry 2025

Supporting information

Table of Contents

Table of Contents 1
1. Materials and instrumentation 2
2. Strains and plasmids 2
3. Media 4
4. Cloning of plasmids 4
5. Transformation of hosts with plasmids 4
6. Cultivation conditions 4
7. Whole cell biotransformations 5
8. H_2 driven whole cell preparative scale reduction of 1a 6
9. Preparation of Cell Wet Weight (CWW) and Cell Dry Weight (CDW) 7
10. HPLC analysis 7
11. Sodium Dodecyl Sulfate- Polyacrylamide Gel Electrophoresis (SDS-PAGE) analysis and immunoblotting 7
12. Scheme 8
13. Figures 8
14. Atom economy and Reaction Mass Efficiency calculations 10
15. Nucleotide sequences 10
16. References 16

1. Materials and instrumentation

All chemicals, buffers and media were purchased from Merck, Sigma Aldrich or Carl Roth. HPLC-MS grade acetonitrile was purchased from VWR. All materials related to molecular biology were purchased from Thermo Fisher Scientific, Promega or New England Biolabs (NEB).

Gases (H_2, O_2, CO_2) for all autotrophic cultivations were purchased from Air Liquide Austria at purities of 5.0 (99.999%) for O_2 and H_2, respectively, and 2.5 (99.5%) for CO_2.

High Performance Liquid Chromatography (HPLC)
HPLC analysis was performed with Shimadzu Nexera UHPLC consisting of the following modules: DGU-20A5 Prominence degasser, LC-30AD pumps, SIL-30AC Nexera autosampler, CTO-20AC Prominence column oven, SPD-M20A Prominence photodiode array detector, LCMS-2020 single quadrupole mass spectrometer equipped with an ESI ionization source and a CBM-20A communications bus module. The data were managed using the LabSolutions software. Data were plotted using R version 4.1.2

Nuclear Magnetic Resonance (NMR)
The NMR spectrum was recorded on a Bruker AVANCE III 300 spectrometer (^{1}H: 300.36 MHz; ^{13}C: 75.53 MHz) with autosampler, Jeol INM-ECZL 400 MHz NMR spectrometer (^{1}H: 399.78 MHz; ^{13}C: 100.53 MHz), and a Varian Unity Inova 500 spectrometer (^{1}H: 499.87 MHz; ^{13}C: 125.69 MHz). Chemical shifts δ are referenced to the residual proton of the deuterated solvent ($CDCl_3$: δ = 7.26 ppm (^{1}H).

2. Strains and plasmids

Table S1. Strains employed in the current study.

Strain	Genotype	Description	Reference/Source
E.coli Top10	*F- mcrA (mrr-hsdRMS-mcrBC*) 80lacZ M15 lacX74 recA1 *ara 139 (ara-leu)7697 galU galK rpsL* (StrR) *endA1 nupG*	Plasmid cloning	Invitrogen
Cupriavidus necator H16	-	Wild type strain	DSM No. 428
Cn02002	*Cupriavidus necator* H16 + pCAT203	Used as negative control. Production of mRFP1 under the control of Pj5 promoter.	[1]
Cn02011	*Cupriavidus necator* H16 + p02013	Used for the production of *Nc*CAR and *Ec*PPTase under the	This work

		control of P_{j5} promoter	
Cn02012	*Cupriavidus necator* H16 + p02014	Used for the production of *Mm*CAR and *Ec*PPTase under the control of P_{j5} promoter	This work
Cn02013	*Cupriavidus necator* H16 + p02015	Used for the production of *Ds2*CAR and *Ec*PPTase under the control of P_{j5} promoter	This work
Cn02015	*Cupriavidus necator* H16 + p02017	Used for the production of *Nc*CAR and *Ec*PPTase under the control of P_{SH} promoter (operon)	This work
Cn02016	*Cupriavidus necator* H16 + p02018	Used for the production of *Nc*CAR and *Ec*PPTase under the control of P_{SH} promoter	This work

Table S2. Plasmids employed in the current study.

**Plasmid**	**Genotype**	**Description**	**Reference/Source**
pCAT203	pBBR1 Rep, pBBR1 OriV, P_{j5}, mRFP1, kan^R	Production of mRFP1	[1]
p02013	pBBR1 Rep, pBBR1 OriV, P_{j5}, *Ec*PPTase, *Nc*CAR, kan^R	Production of *Nc*CAR	This work
p02014	pBBR1 Rep, pBBR1 OriV, P_{j5}, *Ec*PPTase, *Mm*CAR, kan^R	Production of *Mm*CAR	This work
p02015	pBBR1 Rep, pBBR1 OriV, P_{j5}, *Ec*PPTase, *Ds2*CAR, kan^R	Production of *Ds2*CAR	This work
p02017	pBBR1 Rep, pBBR1 OriV, P_{SH}, *Nc*CAR, *Ec*PPTase, kan^R	Production of *Nc*CAR	This work

p02018	pBBR1 Rep, pBBR1 OriV, P_{SH}, *Nc*CAR, P_{SH} , *Ec*PPTase, kan^R	Production of *Nc*CAR	This work

3. Media

Complex media

Lysogeny broth (LB) and Tryptic Soy Broth (TSB) medium were prepared according to the manufacture's protocols. LB composed of: peptone 10 g/L, yeast extract 5 g/L, NaCl 10 g/L. TSB composed of: casein peptone 17 g/L, dipotassium hydrogen phosphate 2.5 g/L, D(+)-glucose 2.5 g/L, sodium chloride 5 g/L, soy peptone 3 g/L.

Defined media

Fructose medium (FN), Fructose-Glycerol–Ammonium (FGN) medium and medium for autotrophic cultivations (AUT) of *C. necator* were prepared according to Lenz et al. 2018. [2]

4. Cloning of plasmids

All *car* and *pptase* genes as well as the promoters P_{j5} and P_{SH} were cloned in the pCAT203 vector using the Gibson assembly method. [3] The constructed plasmids were transformed in *E. coli* Top10 cells. Correct assembly was verified by colony polymerase chain reaction (cPCR). Plasmids from positive colonies were isolated using the GeneJET Plasmid MiniPrep Kit (Thermo Fisher Scientific) and further verified via DNA sequencing (Microsynth).

5. Transformation of hosts with plasmids

E. coli Top10 cells were transformed using the electroporation method. Briefly, an aliquot of electrocompetent cells was transformed with 15 µL of desalted Gibson assembly mix or 100 ng of plasmid DNA. Electroporation was performed in 2 mm cuvettes with an electroporator (Bio-Rad) using the program EC2 (2.5 kV). Electroporated cells were regenerated in 0.5 mL LB medium for 1 h in 37 °C with shaking at 600 rpm.

C. necator cells were transformed using the electroporation method as described by Arhar et al 2024. [4]

6. Cultivation conditions

For plasmid isolation and cryostock preparation, *E. coli* strains were cultivated in 4 mL LB medium containing kanamycin (50 µg/mL). Cultures were incubated overnight at 37 °C with agitation (200 rpm).
For cryostock preparation of the modified *C. necator* strains, were cultivated in 4 mL LB medium containing the antibiotics kanamycin (200 µg/mL) and gentamicin (20 µg/mL).

Cultures were incubated overnight at 28 °C with agitation (200 rpm). Cryostocks were prepared using glycerol (final concentration 20% v/v).

Cultivation of recombinant *C. necator* strains for whole cell biotransformations

Heterotrophic cultivation of recombinant *C. necator* strains producing CARs was performed either in rich medium (TSB) supplemented with 0.6 % w/v fructose or in FGN medium. The cell biomass produced from cultivations in TSB+fructose was used for fructose driven whole cell biotransformations, while the cell biomass produced from FGN cultures was used for H_2-driven biotransformations.

For the **heterotrophic cultivation of *C. necator* strains on rich medium**, a 20 mL preculture (in 100 mL baffled flask) was prepared in TSB medium supplemented with 0.6 % w/v fructose (final pH 7.3), gentamicin (20 µg/mL) and kanamycin (200 µg/mL). The preculture was inoculated with fresh cells from agar plate. The preculture was then incubated overnight at 28 °C with agitation (120 rpm). For the main cultures, 2x1L baffled flasks were used, each one of them filled with 200 mL TSB supplemented with 0.6 % w/v fructose, gentamicin (20 µg/mL) and kanamycin (200 µg/mL). Each flask was inoculated with 6 mL of fully grown preculture. Cultures were incubated at 28 °C/120 rpm until they reached OD_{600} approx. 0.6. Afterwards the cultures were incubated for 19 h at 20 °C with agitation at 120 rpm.

For the **heterotrophic cultivation of *C. necator* strains on FGN** medium the protocol from O. Lenz et al. 2018 was adapted. [2] Two precultures of 75 mL each were set in FN medium supplemented with gentamicin (20 µg/mL) and kanamycin (200 µg/mL). Precultures were set in 300 mL baffled flasks, Fresh cells from agar plate were used to inoculate the precultures, which was then incubated for 19-24 h at 30 °C with shaking (120 rpm). The main culture was prepared in 1 L baffled flask filled with 400 mL of FGN medium supplemented with gentamicin (20 µg/mL) and kanamycin (200 µg/mL). The cell pellet from the preculture was used to inoculate the main culture. For this purpose, the preculture was centrifuged at 4000 rpm/13 min. The main culture was then incubated at 30 °C/120 rpm for 6 days.

For the **preparative scale reduction of 1a**, strain Cn02011 was cultivated on FGN medium. Four precultures of 75 mL each were set in FN medium supplemented with gentamicin (20 µg/mL) and kanamycin (200 µg/mL). Precultures were set in 300 mL baffled flasks. Fresh cells from agar plate were used to inoculate the precultures, which were then incubated for 24 h at 30 °C with shaking (120 rpm). The main culture was set in 2 L baffled flask containing 800 mL of FGN medium supplemented with gentamicin (20 µg/mL) and kanamycin (200 µg/mL). The cell pellet from the precultures was used to inoculate the main culture. For this purpose, the preculture was centrifuged at 4000 rpm/13 min. The main culture was then incubated at 30 °C/120 rpm for 6 days.

7. Whole cell biotransformations

Cell preparation

For the whole cell biotransformations, cells from the main cultures were harvested by centrifugation at 4000rpm/15 min/4 °C. Subsequently, cell pellets were resuspended in 200 mM potassium phosphate buffer, pH 7.4 (10 mL of buffer per 50 mL of cell culture). Resuspensions were centrifuged at 4000xg/15 min/4°C. Cell pellets were resuspended in 200 mM potassium

phosphate buffer, pH 7.4 (0.5 mL of buffer per 50 mL of cell culture). Samples of 0.1, 0.2 and 0.3 mL were acquired in order to measure the cell wet weight (CWW).

Substrate preparation

For the small-scale reactions (1 mL), substrate stock solutions were prepared in a final concentration of 25 mM in 100 mM potassium phosphate buffer, pH 7.0. Due to the low solubility of the compounds **1a** and **1b**, small amounts of 5M KOH were added stepwise to the solution until the substrates were fully solubilized.

For the preparative scale, stock solution of **1a** (50 mM) was prepared in 100 mM potassium phosphate buffer, pH 7.0. In order to fully solubilize the substrate 120 µL of 5M KOH were added stepwise in 50 mL of solution.

Fructose driven whole cell biotransformations

The reactions were conducted in 5 mL glass vials (ND13, Sarstedt) with a standard reaction volume of 1 mL. The reaction mixture consisted of 200 mM potassium phosphate buffer, pH 7.4, cells (26 mg/mL CDW), fructose (0.2% w/v), and substrate (2.5 mM). The reactions were incubated for 16 hours at 28°C with agitation (120 rpm).

H_2 driven whole cell biotransformations

The reactions were conducted in 5 mL glass vials (ND13, Sarstedt) with a standard reaction volume of 1 mL. The reaction mixture consisted of 200 mM potassium phosphate buffer, pH 7.4 and cells (18 mg/mL CDW) and substrate (2.5 mM). The reaction vials with partially open lid were placed in an anaerobic jar filled with a gas mixture of H_2:CO_2:O_2 (7:1:1) (unless stated otherwise). The reactions were incubated for 16 h at 28 °C with agitation (120 rpm).

Reaction termination (adapted from [5])

The reactions were terminated by transferring 0.5 mL of the biotransformation mixture to a 2 mL extraction tube containing 1 mL of methanol. The suspensions were then vortexed for 3 seconds and centrifuged for 25 minutes at 17000xg. The resulting supernatants were transferred to HPLC vials with inlets.

8. H_2 driven whole cell preparative scale reduction of 1a

The preparative scale whole cell biotransformation was performed in a 250 mL shake flask with a final reaction volume of 50 mL. The reaction consisted of the following components: cell biomass from strain Cn02011 (26 mg/mL CDW) resuspended in 200 mM potassium phosphate buffer pH 7.4 and carboxylic acid **1a** (6 mM which corresponds to ~50 mg). The shake flask was placed in an anaerobic jar filled with a gas mixture of H_2:CO_2:O_2 (7:1:1). The reaction was incubated at 28°C with stirring (400 rpm). After 30 h the reaction was centrifuged (4000rpm/15 min) and the supernatant was transferred to a new shake flask. A fresh batch of cells from strain Cn02011 was added to the reaction (26 mg/mL CDW) and the reaction was further incubated for 24 h. Reaction was terminated by centrifugation at 4000rpm/30 min. The supernatant (40 mL) was basified to pH 8 using NaOH (3 M). After lyophilization, the crude yellow solid (210 mg) was purified via column flash chromatography on silica gel 60 (cyclohexane:EtOAc = 2:1) to give **1c** as a white solid (16 mg, yield: 32%). Confirmation of the final product was performed with NMR [^{1}H NMR (300 MHz, $CDCl_3$): δ 6.96 – 6.74 (m, 3H), 5.96 (S, 2H), 4.59 (s, 2H). R_f = 0.30 (cyclohexane/EtOAc 2:1 (v/v)]. Some impurities deriving from the whole cell biotransformation coeluted with the formed product.

9. Preparation of Cell Wet Weight (CWW) and Cell Dry Weight (CDW)

For the determination of CDW, cell pellet from cultures on FGN medium was washed twice with 5 volumes of high purity water. Each washing step was followed by a centrifugation step (17000xg/10 min). After the last washing step, the cell pellet was resuspended in 6 mL of high purity water. Different volumes of the resuspension (0.1 mL, 0.2 mL, 0.3 mL and 0.4 mL) were transferred to 1.5 mL microcentrifuge tubes of determined weight. The tubes were centrifuged at 17000xg/25 min and the supernatant was removed. The total weight of each tube was measured and CWW was determined. Subsequently, the tubes were incubated with the lid open at 60°C for 48 h. Afterwards the CDW was determined.

10. HPLC analysis

Method 1 for analysis of carboxylic acid 1a, aldehyde 1b, alcohol 1c. [5]

HPLC analysis was performed with Shimadzu Nexera UHPLC (details in Materials and Instrumentation) equipped with a column: EC 150/3 NUCLEODUR C18 Gravity, 3 µm (Macherey-Nagel). Analysis was performed with 0.1% formic acid in high purity water (solvent A) and acetonitrile (solvent B). The flow rate of the analysis was 1 mL/min and the column oven temperature was 40 °C. Sample injection volume was 10 µL. Analysis was performed with a stepwise gradient: 0–50 sec 35% B, 50 sec to 2:30 min 70% B, 2:30 min to 2:35 min 90% B, 2:35 min to 3:30 min 90% B, 3:30 min to 3:31 min 35% B, 3:31 min to 4:20 min 35% B. The compounds were detected at 254 nm.

Method 2 for analysis of carboxylic acids 2a-3a, aldehydes 2b-3b, alcohols 2c-3c [6] .

HPLC analysis was performed with Shimadzu Nexera UHPLC (details in Materials and Instrumentation) equipped with a column: EC 150/3 NUCLEODUR C18 Gravity, 3 µm (Macherey-Nagel). Analysis was performed with 0.02% v/v trifluoroacetic acid (TFA) in high purity water (solvent A) and acetonitrile (solvent B). The flow rate of the analysis was 0.7 mL/min and the column oven temperature was 35 °C. Sample injection volume was 10 µL. Analysis was performed with a stepwise gradient: A/B=85:15; 9 min: A/B=40:60; 9.01-9.5min: A/B=20:80. The compounds were detected at 210 nm.

11. Sodium Dodecyl Sulfate- Polyacrylamide Gel Electrophoresis (SDS-PAGE) analysis and immunoblotting

SDS-PAGE analysis was performed in order to confirm soluble protein expression. For this purpose, cells acquired from cultivations in FGN medium were lyzed using the BugBuster™ 10X Protein Extraction Reagent (Novagen) according to the manufacturer instructions. The total protein concentration extracted from each strain was quantified using the Pierce™ BCA Protein Assay Kit (Thermo Scientific™). SDS-PAGE samples containing 10 µg of total protein were separated on SDS-PAGE gels (Bis-Tris, 4-10%, NuPAGE, Thermo Scientific™).

Blotting on a nitrocellulose membrane was achieved via electric current. *Nc*CAR was detected via a conjugated mouse anti-His-HRP antibody (1:1000, 27E8, Cell Signaling Technology). For signal detection, the Clarity Max Western ECL substrate (Bio-Rad) was used in combination with the Syngene® GBox HR16.

12. Scheme

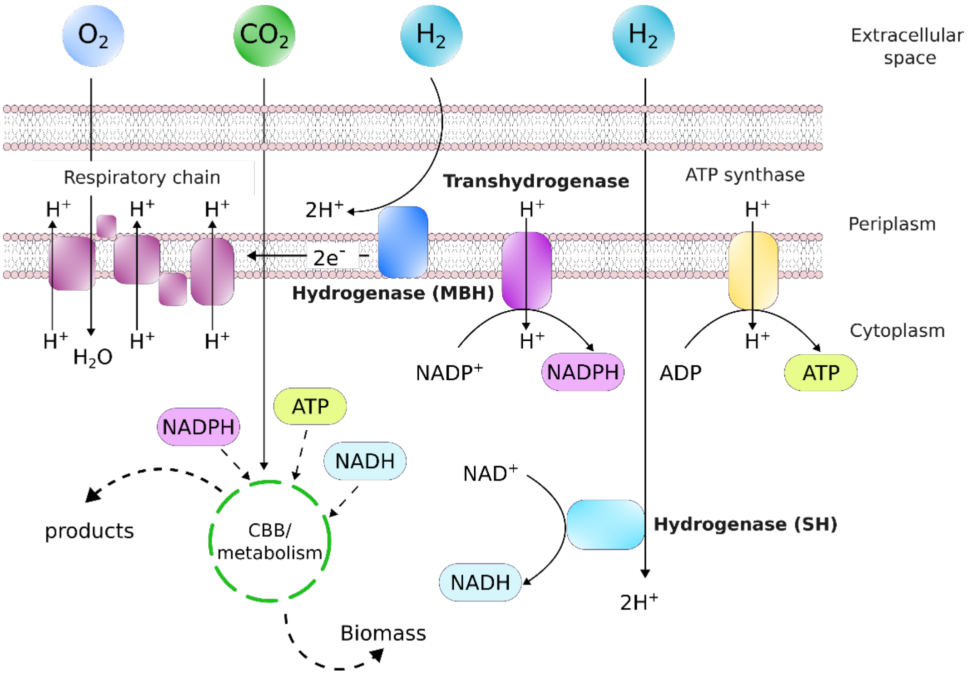

Scheme S1. Simplified overview of *C. necator* metabolic pathway grown under chemolithoautotrophic conditions. Abbreviations: MBH (membrane hydrogenase), SH (soluble hydrogenase). Adapted from Lauterbach et al. [7]

13. Figures

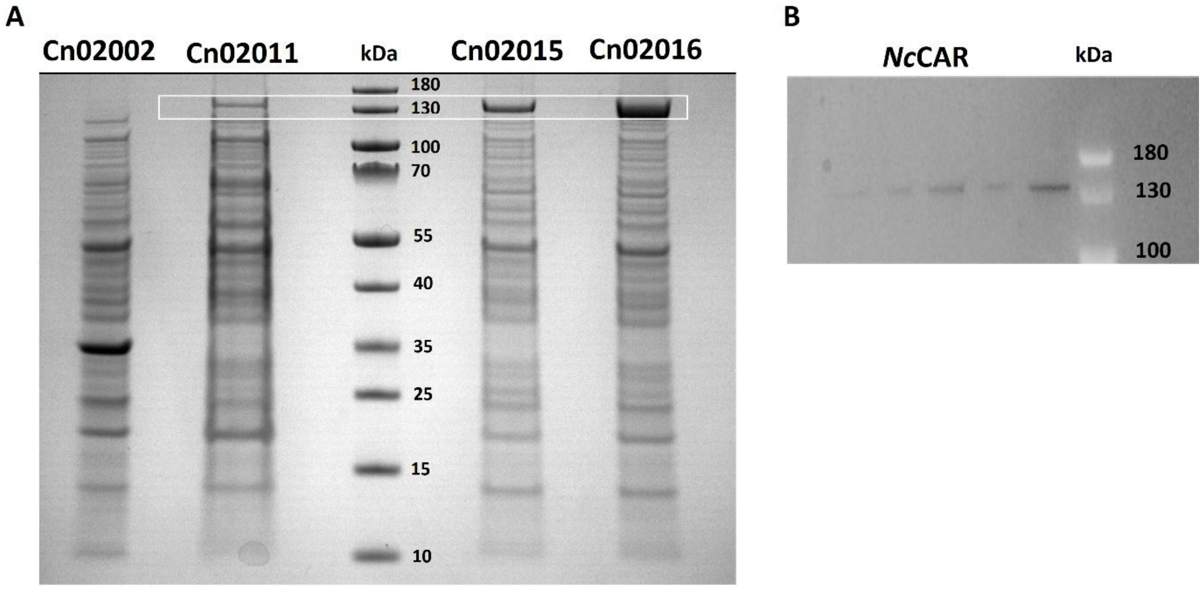

Figure S1: A. SDS-PAGE gel showing the soluble protein extracted from strains cultivated on FGN medium for 7 days. Samples were acquired at the end of cultivation. **Cn02002**: negative control strain (P_{j5}_mRFP1), **Cn02011**: production of *Nc*CAR (P_{j5}_*Ec*PPTase_*Nc*CAR), **Cn02015**: production of *Nc*CAR (P_{SH}_*Nc*CAR_*Ec*PPTase), **Cn02016**: production of *Nc*CAR

(P_{SH}_*Nc*CAR_P_{SH}_*Ec*PPTase). The area marked with a white box indicates the *Nc*CAR (~120 kDa). **B.** Immunoblot detection of *Nc*CAR extracted from strain **Cn02011**.

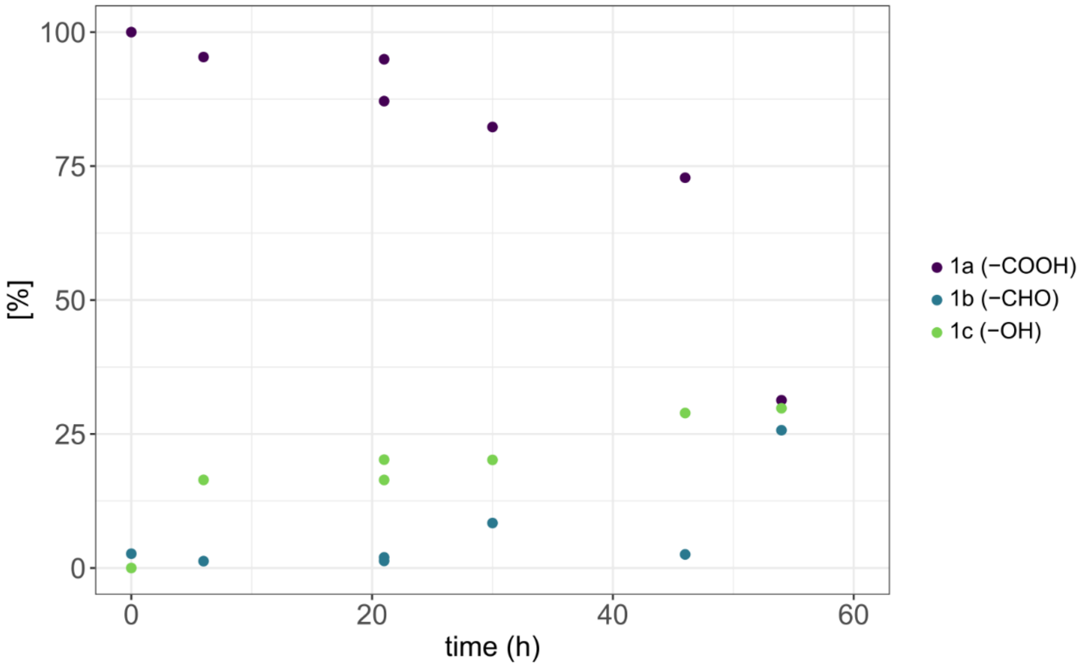

Figure S2: Hydrogen driven whole **cell bioreduction of 1a in preparative scale.** Abundance of substrate **a** (carboxylic acid) and products **b** (aldehyde) and **c** (alcohol) over time. Biotransformations were performed under an atmosphere of H_2:CO_2:O_2 (7:1:1). Conditions: 50 mg **1a**, 26 mg/mL CDW, 28°C, 400 rpm. **[%]** corresponds to remaining substrate and product yields.

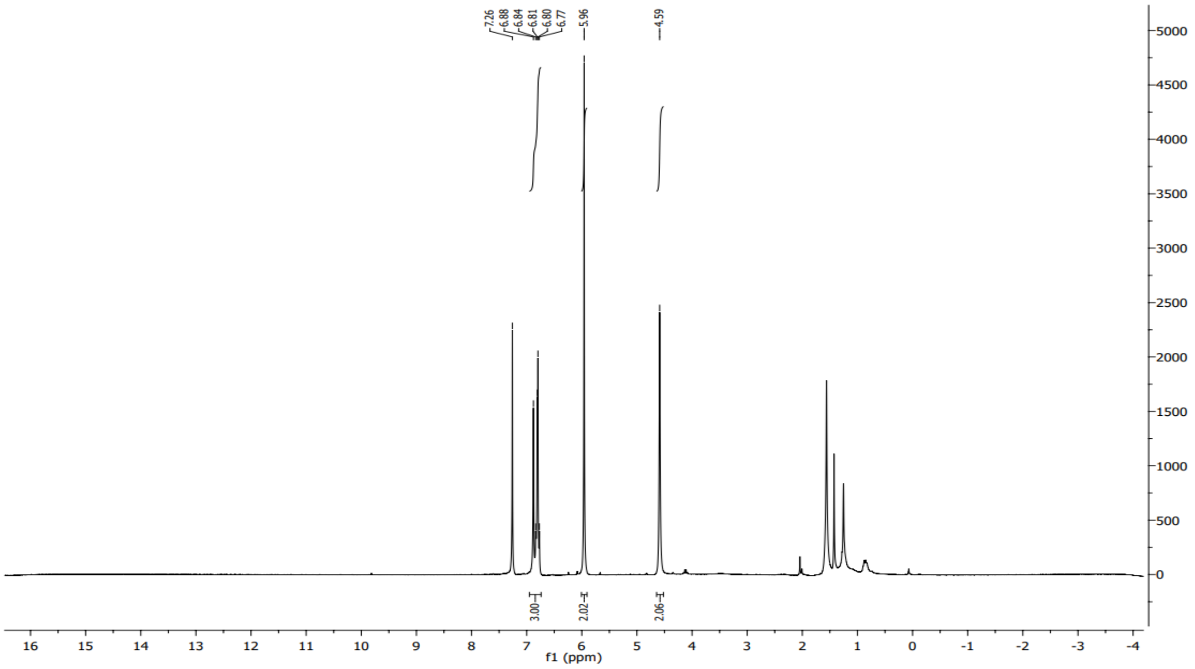

Figure S3: ^{1}H NMR ($CDCl_3$) spectra of isolated compound **1c** from preparative scale synthesis.

14. Atom economy and Reaction Mass Efficiency calculations

Atom economy (AE) is calculated by dividing the molecular weight of the product by the sum total of the molecular weights of all substances formed in the stoichiometric equation for the reaction involved.[8]

$$\text{AE} = \frac{Mr\_\text{product}}{Mr\_\text{Reactant1} + \text{Mr\_Reactant2} + \cdots + \text{Mr\_Reactantn}} x\ 100$$

Reaction Mass Efficiency (REM) is calculated by dividing the mass of the product by the sum of total masses of the reactants .[9]

$$\text{RME} = \frac{mass\ of\ \text{product}}{mass\ of\ \text{Reactant1} + \text{mass of Reactant2} + \cdots + \text{mass of Reactantn}} x\ 100$$

For the reaction **a** we used the calculated RME reported by Constable et al. for the hydrogenation reaction.[9]

For the reaction **b** we estimated the minimum RME assuming that the D-glucose is fully used in the reaction.

For the reaction **c** we hypothesized that molecular hydrogen is consumed in stoichiometric amounts.

Table S3. Calculations of AE and RME.

Reaction	Equation (AE)	AE %	Equation (RME)	RME%
**a**	$\frac{186.34}{200.32 + 32 + 2} x\ 100$	80	Ref: [9]	74
**b**	$\frac{158.20}{172.183 + 1.5x180.16} x\ 100$	36	$\frac{0.0025\ mmol\ x\ 158.2}{0.0025\ mmol\ x\ 172.183 + 0.01\ mmol\ x\ 180.16} x100$	18
**c**	$\frac{138.17}{152.15 + 3x2} x\ 100$	87	$\frac{0.002175\ mmol\ x\ 138.16}{0.0025\ mmol\ x\ 152.15 + 0.0075\ mmol\ x\ 2} x100$	76

15. Nucleotide sequences

Table S4. Elements of plasmid **p02013** used for the development of strain Cn02011 (expression of *Nc*CAR). Sequences were inserted into plasmid vector pCAT203.[1]

**Promoter ($P_{j(5)}$) with RBS (marked)**<br><br>AGCGGATATAAAAACCGTTATTGACACAGGTGGAAATTTAGAATAACTTGTTA GTAAACCTAATGGATCGACCTTAGATCTTTTAAGAAGGAGATATACAT
**Protein: phosphopantetheinyl transferase (*Ec*PPTase) Organism: *E. coli* NCBI: ACA78686.1**

ATGGTCGATATGAAAACTACGCATACCTCCCTCCCCTTTGCCGGACATACGCTG
CATTTTGTTGAGTTCGATCCGGCGAATTTTTGTGAGCAGGATTTACTCTGGCTGC
CGCACTACGCACAACTGCAACACGCTGGACGTAAACGTAAAACAGAGCATTTA
GCCGGACGGATCGCTGCTGTTTATGCTTTGCGGGAATATGGCTATAAATGTGTG
CCCGCAATCGGCGAGCTACGCCAACCTGTCTGGCCTGCGGAGGTATACGGCAG
TATTAGCCACTGTGGGACTACGGCATTAGCCGTGGTATCTCGTCAACCGATTGG
CATTGATATAGAAGAAATTTTTCTGTACAAACCGCAAGAGAATTGACAGACA
ACATTATTACACCAGCGGAACACGAGCGACTCGCAGACTGCGGTTTAGCCTTTT
CTCTGGCGCTGACACTGGCATTTTCCGCCAAAGAGAGCGCATTTAAGGCAAGT
GAGATCCAAACTGATGCAGGTTTTCTGGACTATCAGATAATTAGCTGGAATAA
ACAGCAGGTCATCATTCATCGTGAGAATGAGATGTTTGCTGTGCACTGGCAGAT
AAAAGAAAAGATAGTCATAACGCTGTGCCAACACGAT**TAA**

Sequence upstream of *Nc*CAR containing RBS (marked)

GTTTAACTTTAAGAAGGAGATATACAT

Protein: Carboxylic acid reductase (*Nc*CAR) Organism: *Neurospora crassa* NCBI: XP_955820.1 His tag is marked, TEV cleavage side is highlighted

ATGTCGTACTACCATCACCATCACCATCACGATTACGACATCCCAACGACCGA
AAACCTGTATTTTCAGGGCGCC**ATG**AGCCAACAGCAGAACCCGCCGTATGGTC
GCCGCCTGATCCTGGACATTATTAAAGAACGTGCCCTGAACGAACCGAACCGT
GAATGGGTTTCTGTCCCGCGTAGCTCTGATCCGAAAGACGGTTGGAAAATTCTG
ACCTATCTGGATGCATACAACGGCATCAATCGCGTTGCTCATAAACTGACCCAG
GTTTGTGGTGCCGCGGCACCGGGCAGTTTTCCGACCGTGGCATATATTGGCCCG
AATGATGTCCGTTACCTGGTGTTTGCACTGGGTGCTGTTAAAGCGGGCTATAAA
GCGCTGTTCATCTCAACGCGCAACTCGGCGGAAGCCCAGGTGAACCTGTTTGA
ACTGACCAACTGTAATGTGCTGGTTTTCGATCAGAGCTACAAAGCGACGGTTCA
ACCGTGGCTGCACGAACGTGAAATGACCGCCATTCTGGCACTGCCGGCTGATG
AATGGTTTCCGGCGGATCAAGAAGACTTTCCGTATAATAAAACCTTCGAAGAA
GCCGAATGGGACCCGCTGATGGTGCTGCATACCAGCGGTTCTACGGGCTTTCCG
AAACCGATTGTGGCGCGTCAGGGTATGCTGGCAGTTGCTGATCAATTCCACAAC
CTGCCGCCGCGCGAAGACGGCAAACTGATGTGGATCGTTGAAATGTCGAAACG
TGCAAAACGCCTGATGCATCCGATGCCGCTGTTTCACGCTGCGGGCATGTATAT
TAGCATGCTGATGATCCATTACTGGGATACCCCGGGTGCACTGGGTATTGGTGA
ACGTCCGCTGAGTTCCGATCTGGTTCTGGACTATATCGAATACGCAGATGTCGA
GGGTATGATTCTGCCGCCGGCTATCCTGGAAGAACTGTCACGTGACGAAAAAG
CGATTCAGTCGCTGCAAAAACTGAATTTTGTGAGCTTCGGCGGTGGCAACCTGG
CCCCGGAAGCAGGCGATCGCCTGGTTGAAAACAATGTCACCCTGTGCAATCTG
ATCTCGGCCACCGAATTTACGCCGTTTCCGTTCTATTGGCAGTACGATCAAAAA
CTGTGGCGCTATTTTAACTTCGATACGGACCTGTTCGGTATTGATTGGCGTCTGC
ATGACGGCGAAAGCACCTATGAACAGGTCATCGTGCGCAAAGATAAACACCCG
GGTCTGCAAGGCTTTTTCTACACCTTTCCGGATTCATCGGAATATTCCACGAAA
GACCTGTACAAACGTCATCCGACCCACGAAGATTTTTGGATTTATCAGGGTCGC
GCGGACAATATTATCGTGTTCTCAAACGGCGAAAAACTGAATCCGATTACCATC
GAAGAAACGCTGCAGGGTCATCCGAAAGTGATGGGTGCAGTGGTTGTCGGCAC
GAACCGTTTTCAACCGGCTCTGATTATCGAACCGGTTGAACACCCGGAAACCG
AAGAAGGCCGTAAAGCGCTGCTGGATGAAATTTGGCCGACCGTGGTTCGCGTG
AATAAAGAAACGGTTGCGCATGGTCAGATCGGCCGTCAATATATGGCCCTGAG
CACCCCGGGTAAACCGTTCCTGCGTGCGGGTAAAGGTACCGTGCTGCGTCCGG
GTACCATCAACATGTACAAAGCTGAAATCGATAAAATCTACGAAGACGCAGAA

AAAGGTGTCGCTACGGATGAAGTGCCGAAACTGGATCTGAGCTCTAGTGACGC<br>GCTGATCGTTTCTATCGAAAAACTGTTCGAAACCAGTCTGAACGCGCCGAAACT<br>GGAAGCCGATACCGACTTTTTCACGGCCGGCGTTGATAGTATGCAGGTCATTAC<br>CGCGTCCCGTCTGATCCGTGCGGGTCTGGCCGCAGCTGGCGTTAATATTGAAGC<br>GAGTGCCCTGGCAACCCGTGTCATCTATGGTAACCCGACGCCGAAACGCCTGG<br>CCGATTACCTGCTGAGCATTGTGAACAAAGATTCTAATCAGGGCACCCTGGAC<br>AATGAACATCACGTGATGGAAGCACTGGTTGAAAAATATACCCGTGATCTGCC<br>GACGCCGAAACAGAACAAACCGGCTCCGGCGGACGAAGGTCAAGTCGTGGTT<br>ATTACCGGTACCACGGGTGGCATCGGCTCCTACCTGATTGATATCTGCTCCTCA<br>TCGAGCCGCGTGTCCAAAATTATCTGTCTGAATCGTTCAGAAGATGGCAAAGC<br>GCGCCAGACGGCCTCTAGTTCCGGTCGTGGCCTGAGCACCGACTTTTCTAAATG<br>CGAATTCTATCATGCAGATATGAGCCGCGCTGACCTGGGTCTGGGTCCGGAAGT<br>GTACAGTCGTCTGCTGTCCGAAGTGGATCGCGTTATTCATAACCAGTGGCCGGT<br>CAACTTTAATATTGCGGTGGAATCTTTCGAACCGCACATCCGTGGTTGTCGCAA<br>TCTGGTGGATTTTAGTTATAAAGCCGACAAAAACGTTCCGATTGTTTTCGTCTC<br>ATCGATCGGCACCGTCGATCGTTGGCACGATGAAGACCGCATTGTGCCGGAAG<br>CGTCACTGGATGACCTGTCGCTGGCCGCCGGTGGCTACGGCCAGTCCAAACTG<br>GTGAGCTCTCTGATCTTTGATAAAGCAGCTGAAGTCTCAGGCGTGCCGACCGAA<br>GTCGTGCGTGTTGGTCAGGTTGCGGGTCCGAGTTCCGAAAAAGGTTATTGGAAT<br>AAACAAGAATGGCTGCCGTCGATTGTCGCATCATCGGCTTACCTGGGTGTGCTG<br>CCGGATAGCCTGGGCCAGATGACCACGATCGACTGGACGCCGATTGAAGCCAT<br>CGCAAAACTGCTGCTGGAAGTGTCTGGCGTTATTGATAACGTGCCGCTGGACA<br>AAATCAATGGTTATTTTCATGGCGTTAACCCGGAACGTACCAGTTGGAGCGCTC<br>TGGCCCCGGCAGTCCAGGAATATTACGGTGATCGCATTCAAAAAATCGTTCCG<br>CTGGACGAATGGCTGGAAGCGCTGGAAAAATCTCAGGAAAAAGCCGAAGATG<br>TGACCCGTAACCCGGGCATTAAACTGATCGACACCTATCGCACGTGGTCTGAA<br>GGTTACAAGAAAGGCACGAAATTTGTTCCGCTGGATATGACCCGCACGAAAGA<br>ATATAGTAAAACCATGCGTGAAATGCACGCTGTGACGCCGGAACTGATGAAAA<br>ACTGGTGTCGCCAATGGAACTTT**TAA**

Table S5. Elements of plasmid **p02014** used for the expression of *Mm*CAR. Sequences were inserted into plasmid vector pCAT203.[1]

**Promoter ($P_{j(5)}$) with RBS (marked)**<br><br>AGCGGATATAAAAACCGTTATTGACACAGGTGGAAATTTAGAATAACTTGTTA<br>GTAAACCTAATGGATCGACCTTAGATCT<u>TTTAAGAAGGAGATATACAT</u>
**Protein: phosphopantetheinyl transferase (*Ec*PPTase) Organism: *E. coli* NCBI: ACA78686.1 (as shown above)**
**Sequence upstream of *Mm*CAR containing RBS (marked)**<br><br>GTTTAAC<u>TTTAAGAAGGAGATATACAT</u>
**Protein: Carboxylic acid reductase (*Mm*CAR) Organism: *Mycobacterium marinum* NCBI: WP_012393886.1 His tag is marked, TEV cleavage side is highlighted**<br><br>**ATG**TCGTACTAC<u>CATCACCATCACCATCAC</u>GATTACGACATCCCAACGACCGA<br>AAACCTGTATTTTCAGGGCGCCATGTCA**ATG**TCACCGATTACCCGCGAAGAAC<br>GCCTGGAACGCCGCATTCAAGACCTGTACGCCAACGACCCGCAATTCGCCGCC<br>GCAAAACCGGCGACCGCCATTACGGCAGCAATCGAACGTCCGGGTCTGCCGCT<br>GCCGCAAATTATCGAAACCGTGATGACGGGTTATGCAGATCGTCCGGCACTGG

CTCAGCGCTCCGTCGAATTTGTGACCGATGCCGGCACGGGTCATACCACGCTGC
GTCTGCTGCCGCACTTCGAAACCATTAGTTACGGTGAACTGTGGGACCGCATCT
CAGCGCTGGCCGATGTGCTGTCGACCGAACAGACGGTGAAACCGGGCGATCGT
GTTTGCCTGCTGGGTTTTAACTCGGTTGACTATGCAACCATTGATATGACGCTG
GCTCGTCTGGGTGCAGTCGCTGTGCCGCTGCAAACCTCAGCAGCTATTACGCAG
CTGCAACCGATCGTTGCGGAAACCCAGCCGACGATGATCGCAGCCAGTGTCGA
TGCACTGGCAGACGCAACCGAACTGGCACTGTCCGGCCAAACCGCTACGCGCG
TTCTGGTCTTCGACCATCACCGTCAGGTGGATGCACATCGCGCAGCTGTTGAAA
GCGCTCGTGAACGCCTGGCAGGCTCTGCTGTGGTTGAAACCCTGGCTGAAGCG
ATTGCCCGTGGTGATGTGCCGCGCGGTGCAAGCGCTGGTTCTGCACCGGGTACC
GATGTTAGTGATGACTCCCTGGCACTGCTGATCTATACCTCAGGTTCGACGGGT
GCACCGAAAGGTGCAATGTACCCGCGTCGCAACGTGGCAACCTTTTGGCGTAA
ACGCACGTGGTTCGAAGGCGGTTATGAACCGAGCATTACCCTGAACTTTATGCC
GATGTCTCATGTTATGGGCCGTCAGATCCTGTATGGTACCCTGTGTAATGGCGG
TACGGCATACTTTGTTGCTAAATCAGACCTGTCGACCCTGTTCGAAGATCTGGC
GCTGGTGCGTCCGACCGAACTGACGTTCGTTCCGCGCGTCTGGGATATGGTTTT
TGACGAATTCCAATCTGAAGTTGATCGTCGCCTGGTCGATGGTGCAGACCGTGT
CGCACTGGAAGCACAGGTGAAAGCCGAAATTCGTAATGATGTGCTGGGCGGTC
GCTATACCAGCGCACTGACGGGTAGTGCACCGATCTCCGACGAAATGAAAGCC
TGGGTCGAAGAACTGCTGGATATGCATCTGGTGGAAGGCTACGGTTCTACCGA
AGCAGGCATGATTCTGATCGACGGTGCAATTCGTCGCCCGGCTGTGCTGGATTA
TAAACTGGTGGATGTTCCGGACCTGGGCTACTTTCTGACCGACCGTCCGCACCC
GCGCGGTGAACTGCTGGTTAAAACGGATAGCCTGTTCCCGGGTTATTACCAGCG
TGCGGAAGTGACCGCCGACGTTTTTGATGCAGACGGCTTCTATCGCACGGGTGA
TATCATGGCGGAAGTTGGCCCGGAACAATTTGTCTACCTGGATCGTCGCAACAA
TGTGCTGAAACTGAGTCAGGGTGAATTTGTCACCGTGTCCAAACTGGAAGCGG
TCTTCGGCGATTCACCGCTGGTGCGTCAAATTTATATCTACGGTAACTCGGCGC
GTGCGTATCTGCTGGCCGTTATTGTCCCGACCCAGGAAGCACTGGATGCTGTGC
CGGTTGAAGAACTGAAAGCACGTCTGGGCGATAGCCTGCAGGAAGTTGCGAAA
GCGGCCGGTCTGCAATCTTACGAAATCCCGCGCGATTTTATTATCGAAACCACG
CCGTGGACCCTGGAAAATGGCCTGCTGACGGGTATTCGTAAACTGGCGCGCCC
GCAGCTGAAAAAACATTATGGCGAACTGCTGGAACAGATCTACACCGACCTGG
CACACGGCCAAGCTGATGAACTGCGTTCACTGCGCCAGTCGGGTGCAGATGCT
CCGGTCCTGGTGACCGTTTGCCGTGCAGCTGCAGCACTGCTGGGCGGTAGCGC
ATCTGATGTGCAGCCGGACGCCCATTTTACCGATCTGGGCGGTGACAGTCTGTC
CGCGCTGAGCTTTACGAACCTGCTGCACGAAATTTTCGATATCGAAGTCCCGGT
GGGCGTTATTGTCTCTCCGGCCAATGACCTGCAAGCGCTGGCCGATTATGTGGA
AGCAGCTCGTAAACCGGGTAGCTCTCGCCCGACCTTTGCGAGCGTTCATGGCGC
CTCTAACGGTCAGGTGACCGAAGTTCACGCGGGCGATCTGAGCCTGGACAAAT
TCATTGATGCAGCAACCCTGGCAGAAGCTCCGCGTCTGCCGGCAGCTAATACC
CAGGTCCGTACGGTGCTGCTGACCGGTGCAACGGGTTTTCTGGGCCGTTATCTG
GCTCTGGAATGGCTGGAACGCATGGATCTGGTGGACGGTAAACTGATCTGTCT
GGTTCGTGCGAAATCAGACACCGAAGCGCGTGCCCGCCTGGATAAAACGTTCG
ATTCGGGCGACCCGGAACTGCTGGCCCATTATCGCGCACTGGCTGGTGACCAC
CTGGAAGTGCTGGCGGGTGATAAAGGCGAAGCCGACCTGGGCCTGGATCGTCA
GACCTGGCAACGCCTGGCGGATACGGTCGACCTGATTGTGGATCCGGCGGCCC
TGGTTAACCATGTCCTGCCGTATAGTCAGCTGTTTGGCCCGAATGCGCTGGGTA
CCGCCGAACTGCTGCGTCTGGCACTGACGTCCAAAATCAAACCGTATAGCTAC
ACCTCTACGATTGGCGTGGCGGATCAGATCCCGCCGTCAGCATTCACCGAAGA
TGCTGACATTCGTGTTATCTCAGCGACGCGCGCCGTCGATGACTCGTATGCGAA
CGGCTACTCAAATTCGAAATGGGCGGGTGAAGTGCTGCTGCGTGAAGCACATG

ATCTGTGCGGTCTGCCGGTGGCAGTTTTTCGCTGTGACATGATTCTGGCGGATA<br>CCACGTGGGCCGGTCAGCTGAACGTTCCGGATATGTTTACCCGCATGATTCTGA<br>GTCTGGCAGCTACGGGTATCGCACCGGGTTCCTTCTATGAACTGGCAGCAGAC<br>GGTGCACGTCAGCGTGCTCACTACGATGGTCTGCCGGTGGAATTTATTGCGGAA<br>GCCATCAGTACCCTGGGCGCCCAGTCCCAAGATGGTTTCCATACGTATCACGTC<br>ATGAACCCGTACGATGACGGCATTGGTCTGGACGAATTTGTGGATTGGCTGAAT<br>GAAAGTGGCTGCCCGATTCAACGTATCGCGGATTATGGTGACTGGCTGCAGCG<br>TTTCGAAACCGCACTGCGCGCTCTGCCGGATCGTCAACGCCATAGTTCCCTGCT<br>GCCGCTGCTGCACAATTACCGTCAGCCGGAACGTCCGGTTCGCGGCAGCATTG<br>CACCGACCGATCGTTTTCGCGCAGCTGTCCAGGAAGCAAAAATTGGTCCGGAT<br>AAAGACATCCCGCATGTTGGTGCTCCGATTATTGTCAAATATGTCAGTGATCTG<br>CGTCTGCTGGGTCTGCTG**TAA**

Table S6. Elements of plasmid **p02015** used for the expression of *Ds2*CAR. Sequences were inserted into plasmid vector pCAT203.[1]

**Promoter ($P_{j(5)}$) with RBS (marked)**<br><br>AGCGGATATAAAAACCGTTATTGACACAGGTGGAAATTTAGAATAACTTGTTA<br>GTAAACCTAATGGATCGACCTTAGATCTTTTAAGAAGGAGATATACAT
**Protein: phosphopantetheinyl transferase (*Ec*PPTase) Organism: *E. coli* NCBI: ACA78686.1 (as shown above)**
**Sequence upstream of *Ds2*CAR containing RBS (marked)**<br><br>GTTTAACTTTAAGAAGGAGATATACAT
**Protein: Carboxylic acid reductase (Ds2CAR) Organism: *Dichomitus squalens* NCBI: XP007365347.1 His tag is marked, TEV cleavage side is highlighted**<br>.<br>**ATG**TCGTACTACCATCACCATCACCATCACGATTACGACATCCCAACGACCGA<br>AAACCTGTATTTTCAGGGCGCCCCTTTAACAGCTCACGATCTACCACCCCGCAT<br>TTCTAGCGAATTCCGCATCCCAGAGGATTTAATCCGCCGTGACGACACCGCGCT<br>GCCGCAACTGTACGATTGGAATGCCAAGGAAAACCCGAACTATCCGCTGTTTC<br>TGTACCATGACCCGGCGACCGCACAAAACGAATACATCACCTACTCCACGGCG<br>AATGAGGCGATCAACCGCGCCGCCCGTTACATCACCCACTCTATTGGTAGGGA<br>GCCGAATGCACCAACCGGTTCGCCGGTTGTTGGCATCTTGGCAAATACCGATAC<br>CATTACGTACTTCTGCACCATTATCGGCGCCTTCAGAGCTGGTGTGTGCGCATT<br>CCTGATCAGCACCCGTAACGCCGCGATAGCAGTGGCGGATATGGCAAAGCGTA<br>CCGGCCTTACCCACATCATCGTGAGCCCGGACGCGATCATGAACGAGATCGCC<br>GATGGTGCCATAAAGCTGTTGGCTTCCGATGGGGTTAATGTTCAGAAACATCGT<br>ATGCCGGTGTTTGAAGACCTGTTCCCGGCGACGCCGATGGCGGATAGCCTGTTC<br>GAAAAGGCGGTGGAGCTGCCGAAAACTTACGACGTGCGTGCGTACAATACCAT<br>CATGCATAGCAGCGGTTCTACGGGACATCCGAAGCCGATCCGTTGGACCATTA<br>AAGGTATTCTGTCTTGGGGTCAAGAACCGTTGAAGTGCAATGTAGACATCAAA<br>GGCACGATCATGGGCGCGCATGGTAGCCCGATGTTCCACGGCCTGGGCTCGTT<br>CTTGTACTCCGCTGCGCCGATTAACGGTTTCATCGTTGCGGCGTTTAAGCCGGC<br>GAGCCCACCGACGGTCCCGACCCCGGACGCCGTTTGGACCGGCATTGCTGCGA<br>CCGGCAGCGACTTCAGCTGGAGCGTGCCGAGCTTTATCGAAGAGTGGGGTCGT<br>GATCCGGAAAAAGTGCTGTATATGAAGCGCATGCGTGGTGTGATCTTTGGCGG<br>TGCGGCGTTGAATGATCAGGTTGGTAATACTCTGGCGGCTCAAGGTGTGAGCCT<br>ATACACCGTGTATGGTTGTACGGAGGTCGGCTTGATTAATACCTTTGCTCGCCC<br>GAATCCGGGTATGGATTGGGCATATTGGTCCGTGACCGCGAGCCTAAAAGGTA

GTTTCCGCCCGATGGGCGACGGCACCTACGAGGTTGTGGTGCTGAGCCCGCCG
GACTTACCGCTGCCGGTGACCAATACCAAAGTCGGTGACCAGGATGCTTACGC
TACCAGCGATCTGGCGGTACCACATCCGACGCAGCCTGGTCTTTGGAAGATTGT
GGGCCGTGCTGACGAGCAGATTATTCTCTCCAACGGTGAAAAGACGAACCCCG
TTCCACTGGAAAAGATGATCAACGAGGACCCGTTTGTGAAAAGCAGCGTCATG
TTCGGTCGTGGCAAATTTCAGAACGGCGTCCTGATTGAACCGACTGAAGACTTT
CAAATTGACCCGACTGATGCGAAACAACTGGAGGCGTACCGCAACAAAATCTG
GCCTACAATCGAACGCGTGAATGAGTTCGCACCGCAACACAGCCGTATTTTTA
AAGAAATGATCCTGGTTACCCACCCGAGCAAACCGTTCGAGCATAACGCGAAG
GGCCTGCCGCGTCGTGGTGTAATTCTGAAGGAGTATGCAGATGAAATTGAAGC
TCTGTACAAAGAGGTTGAAAACAGCGCCCAGAGCGAGTTTGCGCCGCCGGCGG
TTTGGGATCCAGCCTCCACGTTGGCGTTCGTGCGTACCGTTGTTCAGTCCACCC
TGCGTCGTGCAATCGCTGACGACGCGGATATTTTTCGTAATGGCGGTGATTCGC
TGCAGAGCACCTGGATTCGCAACACCTTGTTGCGTGCGATTCGTGAAACCCACA
AAGATGCCGCGGTTCGTCTGCCGATGAATGTCGTCTTCCAGGCTCCGACCATCG
CAAGCCTGGCCACGCTCTTGCACAACATCATCCACAGCGCTGAAGAGGCGGTA
CGTGCGCCGCAGGACCTGTGGAAACACGTGGAGAAGTATTCTGCCAATCTGCC
GGCTCGCCCTGCGAACCTGGTCGAACGTCCCGAGGGCCAGAAAGATGTTGTCA
TCATCACCGGCACGACCGGAGGTTTCGGCTGCGACACCCTTGAGCACCTGTTGC
GTGATGAGACAGTGGAACGTGTTTATGCCTTCAACCGTAAGGGCGCGCAAGCG
TTAGGCCGTCAGCGTAAGCAGTTTGAGGCTCGTGGTTTGGACGTTTCTTTACTT
GACAGCCCTAAGTTCCGTATGGTTGAAGCGGTGTTGCATGAACCTGGCTTCGCG
GTGGAGGCTGAACTGCTCGACGAGATCCGCACATCAGCAACCCATATTCTGCA
CAACGCTTGGAAAGTGGACTTTAACCTGTCTATCGCTTCTTTCGAGCACGATAT
CCAGGGCGCCCGCAACCTGGTCGACTTGGCGATTTCGTCGCCGTATAAAAAG
CGCCAACCGTTGTATTCGTTTCCTCTGTGGGGGTGTTTTCTGGTTGTAAAATTTC
TCCGCCTGTTCCGGAGATCCCGTTGGACGACCCGGCGTCCGCTTTCGGCACGGG
TTATAGCGAGAGCAAATGGGTTACCGAGCACGTTCTGCAAAACGTTACTGAAC
GGACTGGGGTGCATACCGTGGTTATGCGTCTGGGCCAGGTTGCGGGTGACCGT
AAGGGTTATTGGAACGAGCGTGAATGGTTCCCGTCCTTGGTGAAGAGCGCATT
GTTCCAAAAGTGCCTGCCGGAGCACGACGGCAAGGTGACCTGGTTTCCGGCGT
ACGAAGCGGCTAAGGCCTTTGCGGAAATGAGACGCTCGCCAGAGTCTGTTCTG
CACCTGGTGCACCCGCACCCGGTGCCGTGGCATGCACTCCTCGCACCGATTGCG
GAAGAACTGGGCGTTCCACTCGTTCCGTATACGCAATGGCTGGAAAAGCTGGA
AGGTAGTGTCGAGCACGGCTCCGCTGAAGAAGTTGAAGCAATGAAGGCGAACC
CGGCTCTGCGTCTGTTGCCGTTTTACAAAGCCCAAGCCGGTACCATGACCCCGG
ATCGTGAGGCCATGGGTTTGGTGTTCATCTCGACTGAGAAAGCGGTTCGTGTTT
CCGAGTCCCTGGCGAAGATGCCGCAACTGGGTGCTGAGCGCGCACGCATGTGG
CTGGCGGCATGGAAACGCAGCGGTTTTATTCAT**TGA**

Table S7. Elements of plasmid **p02017** used for the development of strain Cn02015 (expression of *Nc*CAR). Sequences were inserted into plasmid vector pCAT203.[1]

Promoter region (P_{SH}) with RBS (marked)

CTCATCGGGTCCTGCTCATAGGTTCGTAGCCGCGATCGCCAACCAAAAAAACC
CTCTCCTGCGGGAAATCCGCACGCTACGTTCTGTGGGAACCGGAGGCGGGTGA
CTGCCTCCGGTCACCCGGTGCTCGGGGTGCGATTCCCCGGGTCTACTTACCAAA
TCGGCCGCGCACCCAATGAGAGGCGCTGGCACAAGCTTGCACAGACTTGCCCG
CCAAGCGGAAGCAGCCTTGCCACATCGGCCGACCCAATGGCAATGCCGCTGCC
ACCCGCCGGATGGCCGTTCTGGAAACGGCTTGAGCGACGTCAAGAATTTCCTTT

CTCGACAAGCACTTAGCCGGGCCTCCTGGTGGTTTCCCTTAGGCCCTGCGAAAT<br>TGGCGCACATCCTGCGTTCCACCTGCGCATCGAAGTGACGCACCAAGCAAGGG<br>GCGAACATTAGTAAGGAGGAGACAAC
**Protein: Carboxylic acid reductase (*Nc*CAR) Organism: *Neurospora crassa* NCBI: XP_955820.1 (as shown above)**
**Sequence upstream of *Ec*PPTase containing RBS (marked)**<br>GGATCCGTTTAACTTTAAGAAGGAGATATACA
**Protein: phosphopantetheinyl transferase (*Ec*PPTase) Organism: *E. coli* NCBI: ACA78686.1 (as shown above)**

Table S8. Elements of plasmid **p02018** used for the development of strain Cn02016 (expression of *Nc*CAR). Sequences were inserted into plasmid vector pCAT203.[1]

**Promoter region (P_{SH}) with RBS (marked)**<br><br>CTCATCGGGTCCTGCTCATAGGTTCGTAGCCGCGATCGCCAACCAAAAAAACC<br>CTCTCCTGCGGGAAATCCGCACGCTACGTTCTGTGGGAACCGGAGGCGGGTGA<br>CTGCCTCCGGTCACCCGGTGCTCGGGGTGCGATTCCCCGGGTCTACTTACCAAA<br>TCGGCCGCGCACCCAATGAGAGGCGCTGGCACAAGCTTGCACAGACTTGCCCG<br>CCAAGCGGAAGCAGCCTTGCCACATCGGCCGACCCAATGGCAATGCCGCTGCC<br>ACCCGCCGGATGGCCGTTCTGGAAACGGCTTGAGCGACGTCAAGAATTTCCTTT<br>CTCGACAAGCACTTAGCCGGGCCTCCTGGTGGTTTCCCTTAGGCCCTGCGAAAT<br>TGGCGCACATCCTGCGTTCCACCTGCGCATCGAAGTGACGCACCAAGCAAGGG<br>GCGAACATTAGTAAGGAGGAGACAAC
**Protein: Carboxylic acid reductase (*Nc*CAR) Organism: *Neurospora crassa* NCBI: XP_955820.1 (as shown above)**
**Promoter region (P_{SH}) with RBS (as shown above)**
**Protein: phosphopantetheinyl transferase (*Ec*PPTase) Organism: *E. coli* NCBI: ACA78686.1 (as shown above)**

16. References

1. C. C. Azubuike, A. M. R. Gatehouse and T. P. Howard, *New Biotechnol.*, **2021**, *65*, 20–30.
2. O. Lenz, L. Lauterbach and S. Frielingsdorf, in *Methods in Enzymology*, ed. D. Zamble, Academic Press, 2018, vol. **613**, pp. 117–151.
3. D. G. Gibson, L. Young, R.-Y. Chuang, J. C. Venter, C. A. Hutchison and H. O. Smith, *Nat. Methods*, 2009, ***6***, 343–345.
4. S. Arhar, T. Rauter, H. Stolterfoht-Stock, V. Lambauer, R. Kratzer, M. Winkler, M. Karava, R. Kourist and A. Emmerstorfer-Augustin, *Microb. Cell Fact.*, 2024, ***23***, 1–13.
5. D. Goj, S. Ebner, M. Horvat, S. Arhar, L. Martínková and M. Winkler, *J. Biotechnol.*, 2024, ***382***, 44–50.
6. G. A. Strohmeier, I. C. Eiteljörg, A. Schwarz and M. Winkler, *Chem. Eur. J.*, 2019, ***25***, 6119–6123.
7. L. Lauterbach and O. Lenz, *Curr. Opin. Chem. Biol.*, 2019, ***49***, 91–96.

8. R. A. Sheldon, *Green Chem.*, 2017, ***19***, 18–43.
9. D. J. C. Constable, A. D. Curzons and V. L. Cunningham, *Green Chem.*, 2002, ***4***, 521–527.
